# Congenital Morphinism, with Report of Cases*Read before the Southern Medical Association, Hattiesburg, Miss.; also before the Tri-State Medical Society, Memphis, November, 1911.

**Published:** 1912-03

**Authors:** Geo. E. Petty

**Affiliations:** Memphis, Tenn.; Member Memphis and Shelby County Medical Society, Tenn., State, Tri-State, Mississippi Valley, Southern and American Medical Associations


For Texas Medical Journal.

### Congenital Morphinism, With Report of Cases.*

BY GEO. E. PETTY, M. D., MEMPHIS, TENN.

Member Memphis and Shelby County Medical Society, Tenn., State, Tri-State, Mississippi Valley, Southern and American Medical Associations.

Congenital morphinism is a rather rare condition. Comparatively few opium-using mothers conceive and fewer still of the children born of such mothers live beyond the third day after birth. This high mortality among the infants of such mothers is not unavoidable, but, managed as they usually are, a large majority of children born to such mothers die on the second or third day after birth. This is due to the fact that the child's blood and tissues are as fully saturated with the narcotic as are those of its mother; in fact, so far as the physical elements of the addiction are concerned, the child is as much an habitue as is its mother. Severance of the placental circulation through which the child has been receiving the narcotic shuts off that supply and, if the drug is not administered to the child, it suffers the shock and collapse incident to the abrupt withdrawal of opiates from an habitue. Few adults are able to stand such a strain; therefore, a newly-born infant could not be expected to do so. Some infants born under such conditions, however, are managed so as to preserve their lives and a few of them have come under my care. Note the following cases:

In May, 1901, W. T. B. and wife came for treatment for morphinism, bringing two children with them; one a boy of three years, the other a girl one year of age. The mother was using morphine before the first conception, and when the baby was born it was fortunately under the care of a physician who realized the danger of the sudden withdrawal of an opiate from such an infant, and he gave paregoric in full doses for the first three days, and then the baby was put to the breast and allowed to get its drug supply in that way. When this child was a year old an effort was made to wean it, but it was very fretful and suffered so severely that the mother says that she did not have the heart to deny it the breast, and it was allowed to continue to nurse. Notwithstanding this, however, and notwithstanding the fact that she continued to use the opiate and that menstruation was absent, she conceived again and the girl was born two years after the boy.

*Read before the Southern Medical Association, Hattiesburg, Miss.; also before the Tri-State Medical Society, Memphis, November, 1911.

This child was managed in the same way as the first for the first few days after birth, and then both children were allowed to nurse. They both received their narcotic through the milk, and the mother had not succeeded in weaning either of them. Therefore, they were brought with her for treatment.

The mother and children were prepared for the withdrawal of the opiate by active elimination, the opiate was discontinued, and they were given my usual treatment, the children taking the same remedies as the mother, but in relatively smaller doses. The children stood the treatment as well as the mother and all were brought out in good condition in a short time. Neither of the children were allowed to nurse after the treatment. The mother was much emaciated from the drain of nursing the two children, but her convalescence was rapid after the children were weaned and both the mother and the two children have remained free from their addiction. The children are healthy and vigorous and show no evidence of their former addiction. It has now been a little over ten years since these cases were treated.

In November, 1909, a Texas physician wrote, asking advice as to the management of his wife, who he said was addicted to the use of morphine and was about seven months pregnant. Not being able to find anything in medical literature to guide him in the management of such a case, he wished me to give him such aid as I could. Had the patient been near me, I would have taken her off of the drug, even at that advanced stage of pregnancy, but a long railroad journey for such a patient made another course more expedient.

I wrote him that I thought we would be able to carry his wife through her confinement without complication, and advised that the use of the opiate be continued without any effort at reduction. I also called his attention to the importance of keeping up active elimination, both by bowels and kidneys during the remainder of gestation, and suggested suitable remedies for that purpose, to the end that the patient's system be kept in the best condition and be as nearly free from toxic matter as possible at the time of her confinement. The only complications likely to occur being of toxic origin.

These suggestions were faithfully carried out, and on December 30th she was delivered of a healthy child, at full term, in a normal labor. Six hours after the delivery, the child began to show signs of restlessness and discomfort, and following the plan I had outlined it was given a drop of laudanum, and this was repeated in half an hour. This did not give relief, and one hour later two drops were given at one dose. This gave relief and the baby was quiet for several hours, but when it began to show signs of discomfort again three drops of laudanum were given at one dose. This proved to be a fairly effective dose, and a dose of this size was given every six hours for the next two days. By this time the milk flow was fairly well established, but the supply was not abundant and the child did not get as much narcotic through the milk as it had been accustomed to before birth, and it was found necessary to supplement it by the administration of two drops of laudanum three times a day in order to keep the child free from abstinence symptoms. When the child was a few weeks old an effort was made to discontinue these doses and depend upon the supply from the milk alone, but the child was very fretful and restless when these doses were not given, so it was thought best to continue them until the mother and child could be sent for treatment. This was done when the child was two months old.

Upon admission, the mother was found to be using about ten grains morphine daily; the infant was nursing and receiving in addition to the quantity it obtained from the milk from six to nine drops of laudanum daily. Both were prepared for withdrawal by thorough elimination and the drug discontinued at once in both cases. The mother was kept under the influence of hyoscine to a moderate degree for a couple of days and the child was allowed to continue to nurse. Within six hours from the time the administration of hyoscine to the mother began the child showed distinct signs of the effects of that remedy, but it was fretful and manifestly uncomfortable. At this time a dose of 1-1000 grain hyoscine was given to the baby, and this dose was repeated every four to six hours for the next thirty-six hours. By the end of the third day from the withdrawal both patients were fairly out from under the influence of hyoscine and all other remedies and were comfortable. On the fourth day their condition was still more satisfactory, and from that time on their convalescence was normal and uninterrupted. Neither of them suffered during the treatment to such a degree as to show it.

The doctor—the husband and father—writes under date of November 4, 1911, saying that both mother and baby are in good health and free from their addiction. He sends a picture of the little girl, who is now a hearty, robust child of two years.

Every child born of an opium-using mother should be given an opiate for the first three days after birth. After that the milk may be depended upon to contain enough of the drug to satisfy the demands of the child's system, or, if it is thought best not to allow the child to nurse, the administration of the opiate should be continued until such time as the child is strong enough to stand the withdrawal. By this means their lives can be preserved. The results obtained by two mothers who have come under my care illustrates this point.

Mrs. M. J. came for treatment in May, 1910. She had been taking McMunn's Elixir of Opium thirty-one years; is the mother of eighteen children; only the first two and the last one lived beyond the third day after birth. The first two children were born before she became addicted to the opiate. During the third pregnancy she was threatened with miscarriage, and McMunn's Elixir of Opium was given to prevent it. During the last half of the period of gestation the opiate was administered to her two or three times a day, and, while it served the purpose of preserving the pregnancy, it also fastened the opium habit upon her, and she was not able to throw it off. The child was fully developed and apparently perfectly healthy, but on the day following its birth it began to show signs of illness, and it grew progressively worse and died forty-eight hours after delivery in convulsions. Neither the physician in attendance or the mother understood the necessity of giving the child an opiate, and none was given. The death was attributed to unknown causes.

This pregnancy was followed by another within fifteen months, in which she was confined under the same management, with death of the child within three days after birth, and this pregnancy by another and another, until this woman had given birth to fifteen while using an opiate habitually. All the children died within three days from their birth, most of them dying by the end of the second day. The same physician attended her in all these confinements, and both the mother and the physician looked upon the death of the children as a neutral consequence of the mother's condition, and inevitable.

In her last confinement she was attended by another physician, who appreciated the conditions under which the child was being brought into the world and understood the necessity of protecting it from the shock incident to the abrupt withdrawal of the opiate. In order to do this he gave paregoric in ten drops and these were repeated at such intervals as was found necessary to keep the child comfortable and free from abstinence symptoms.

The child was not allowed to nurse, but the paregoric was kept up for a few months and then gradually discontinued. This child is now a stout, healthy boy of thirteen years, and the mother is firm in her belief that, had the same course been pursued with the other children, they would have lived also. They were all well developed and apparently normal children and showed no signs of illness or discomfort during the first twelve hours after birth, but from that time on their illness grew rapidly and they either went into complete collapse or died in convulsions by the end of the third day after birth.

A case which I now have under treatment gives a history which in some particulars is very similar to the above, while in others it differs greatly. This woman—Mrs. C. J. D.—has been using morphine by the mouth forty-five years; during most of that time has taken with regularity twenty grains morphine per day. She was married in 1863; first child in 1864; premature at sixth month of pregnancy. A second pregnancy soon followed, which resulted in miscarriage at third month. The third pregnancy occurred in 1866. Miscarriage was threatened at second month and morphine was administered to preserve the conception. This was used occasionally to relieve symptoms for the first few months, but before gestation was complete the drug had been used daily for more than three months, by which time the morphine addiction was established, but she was carried to full term and delivered of a normal child. The mother says that either from intuition or upon the advice of the midwife who attended her paregoric was administered to the child when it became fretful, and this was kept up for several months before it was entirely discontinued; the child lived to be a grown woman.

After the mother's recovery from this confinement an effort was made to discontinue the use of the morphine, but without success, and its use has been continued from that time until now. As she did not allow this child to nurse, another pregnancy occurred within a year and this was followed by others in rapid succession until eleven children had been born to this morphine-using mother. She went to full term with all of them, and none of the children died in infancy; six of them are now living and none of them show peculiarities which could be attributed to the condition of the mother at the time of their birth.

The same course was pursued in the case of the last ten children as in that of the first one that lived. An opiate was administered to them on the day following their birth, usually about twelve hours after birth. The mother says that about twelve hours after birth they began to show signs of discomfort and, as she understood what these symptoms meant, she administered an opiate, and this was repeated at such intervals as was found necessary, and, after the babies were a few months old, the opiate was discontinued by gradual reduction, and this was usually done without very much difficulty. None of the children were allowed to nurse.

One unique feature of this case is that no menstrual flow has occurred since the third pregnancy, the ten conceptions following that having occurred without the appearance of menstruation at all. As a rule, the habitual use of opiates arrests menstruation, but from these cases it is quite evident that it does not always arrest ovulation, if indeed it does so at all. This woman passed the menopause without knowing when it occurred.

### CONCEALED MORPHINISM IN PARTURIENT WOMEN.

When a child is born of a woman whose habits are not known and the child appears to be healthy and normal in every respect at birth, but in the course of twelve to eighteen hours afterwards begins to show signs of discomfort, the symptoms taking the form of gastro-intestinal disturbances with embarrassed portal circulation and these symptoms become progressively more noticeable from hour to hour, concealed narcotic addiction in the mother should be suspected and evidence of the existence of such condition diligently searched for. Narcotic addiction in the mother will often be found to explain this sudden, and otherwise unaccounted for, illness of the infant. When this condition of affairs is suspected, although the mother may not admit it, it is well to give the child an opiate, say, one drop of laudanum. If this shows less than the normal effect, repeat it in half an hour, watch for effect again and, if not seen, repeat until the child is brought fairly well under the influence of the opiate or until it is restored to the apparently normal condition which existed for the time immediately succeeding its birth. If the child is found to have an undue tolerance for the opiate, or if its effects restore an apparently normal condition instead of inducing narcotic sleep, the existence of narcotic addiction may be accepted as a fact and the case handled accordingly. If it is a narcotic case, as the effects of the narcotic administered begins to wane, the symptoms of distress will again make their appearance, and nothing but a narcotic will relieve them and this should be given as in cases where addiction is admitted. Few mothers, however, will be found to stubbornly persist in denying their real condition when they are made to realize that the life of their child may depend upon their making their condition known at least to the attending physician.

---

If the surgeon desires to discover carcinoma of the cervix in a curable stage, women past middle life must be examined periodically, for to wait until symptoms appear is often to discover the disease too late.—*American Journal of Surgery.*

---

Presentation of a Portrait of Dr. Wyeth.—At the meeting of the New York Academy of Medicine on February 14th, Dr. Simon Baruch, as chairman of the committee designated to procure a portrait of Dr. Wyeth, presented the portrait, which was painted by J. Campbell Phillips. In making the presentation Dr. Baruch read letters from several contributors to the fund expressive of the high esteem in which Dr. Wyeth is held. The picture is to be placed in the gallery devoted to portraits of presidents of the New York Academy of Medicine.

---

Perforation of Gravid Uterus.—S. Wiener reports a case of this condition and discusses its diagnosis and treatment. The course that should be pursued as soon as perforation of the uterus is recognized is as follows: All further manipulation should cease, intrauterine irrigation should be dispensed with; the uterus and vagina should be packed; the patient placed in the Fowler position, and further developments awaited. Barring peritoneal infection, the majority of cases will show no further ill effects. If the uterus has not yet been completely emptied, this may be undertaken at a future time. However, if the accident has not been at once recognized, if there has been intraperitoneal manipulation with the curette or placental forceps, or if omentum or intestine has actually been drawn down into the vagina an exploratory laparotomy is urgently indicated. This should be performed just as soon as proper preparations can be made. A few hours may decide the question of the life or death of the patient. To await the development of active symptoms of peritonitis or the collapse supervening upon intra-abdominal hemorrhage may turn the scale in favor of a fatal issue. The exact method of procedure after opening the abdomen must be determined by the conditions met with.—*Medical Record.*

